# The crosstalk between neuropilin-1 and tumor necrosis factor-α in endothelial cells

**DOI:** 10.3389/fcell.2024.1210944

**Published:** 2024-06-27

**Authors:** Ying Wang, Enfeng Wang, Mohamed Anany, Simone Füllsack, Yu Henry Huo, Shamit Dutta, Baoan Ji, Luke H. Hoeppner, Sreenivasulu Kilari, Sanjay Misra, Thomas Caulfield, Craig W. Vander Kooi, Harald Wajant, Debabrata Mukhopadhyay

**Affiliations:** ^1^ Department of Cardiovascular Medicine, Rochester, MN, United States; ^2^ Department of Biochemistry and Molecular Biology, Mayo Clinic, Rochester, MN, United States; ^3^ Department of Biochemistry and Molecular Biology, Mayo Clinic, Jacksonville, FL, United States; ^4^ Division of Molecular Internal Medicine, Department of Internal Medicine II, University Hospital Würzburg, Würzburg, Germany; ^5^ Department of Microbial Biotechnology, Institute of Biotechnology, National Research Centre, Giza, Egypt; ^6^ Department of Cancer Biology, Jacksonville, FL, United States; ^7^ The Hormel Institute, University of Minnesota, Austin, MN, United States; ^8^ Masonic Cancer Center, University of Minnesota, Minneapolis, MN, United States; ^9^ Department of Radiology, Mayo Clinic, Rochester, MN, United States; ^10^ Department of Neuroscience, Mayo Clinic, Jacksonville, FL, United States; ^11^ Department of Biochemistry and Molecular Biology, University of Florida, Gainesville, FL, United States

**Keywords:** tumor necrosis factor-α, neuropilin-1, endothelial cells, inflammatory response, receptor

## Abstract

Tumor necrosis factor-α (TNFα) is a master cytokine which induces expression of chemokines and adhesion molecules, such as intercellular adhesion molecule 1 (ICAM-1) and vascular cell adhesion molecule 1 (VCAM-1), in endothelial cells to initiate the vascular inflammatory response. In this study, we identified neuropilin-1 (NRP1), a co-receptor of several structurally diverse ligands, as a modulator of TNFα-induced inflammatory response of endothelial cells. NRP1 shRNA expression suppressed TNFα-stimulated leukocyte adhesion and expression of ICAM-1 and VCAM-1 in human umbilical vein endothelial cells (HUVECs). Likewise, it reduced TNFα-induced phosphorylation of MAPK p38 but did not significantly affect other TNF-induced signaling pathways, such as the classical NFκB and the AKT pathway. Immunofluorescent staining demonstrated co-localization of NRP1 with the two receptors of TNF, TNFR1 and TNFR2. Co-immunoprecipitation further confirmed that NRP1 was in the same protein complex or membrane compartment as TNFR1 and TNFR2, respectively. Modulation of NRP1 expression, however, neither affected TNFR levels in the cell membrane nor the receptor binding affinities of TNFα. Although a direct interface between NRP1 and TNFα/TNFR1 appeared possible from a protein docking model, a direct interaction was not supported by binding assays in cell-free microplates and cultured cells. Furthermore, TNFα was shown to downregulate NRP1 in a time-dependent manner through TNFR1-NFκB pathway in HUVECs. Taken together, our study reveals a novel reciprocal crosstalk between NRP1 and TNFα in vascular endothelial cells.

## Introduction

Induced expression of cell-surface adhesion molecules, including vascular cell adhesion molecule 1 (VCAM-1), intercellular adhesion molecule 1 (ICAM-1), and endothelial leukocyte adhesion molecule (E-selectin), is a major feature of endothelial cell activation and it promotes leukocyte recruitment, vascular permeability, clotting and antiviral response in diverse pathological processes (Szmitko et al., 2003; Pober and Sessa, 2007; Liao, 2013; Jin et al., 2020). The pleiotropic effects of tumor necrosis factor-α (TNFα), one of the major pro-inflammatory cytokines, on vascular endothelial cells have been extensively studied (Pober, 2002; Zhang et al., 2009; Jeucken et al., 2019). As a result, intensive studies have been performed to reduce TNFα levels and its downstream signaling pathways to improve endothelial cell function. Although anti-TNFα therapies have revolutionized the management of autoimmune diseases, they have demonstrated controversial effects in cardiovascular functions (Rolski and Blyszczuk, 2020; Singh et al., 2020). Thus, a deeper understanding of TNFα-stimulated signaling pathways offer the chance to identify specific targets for the inflammatory process affecting vascular endothelial cells.

Neuropilin-1 (NRP1) is known as a co-receptor of several structurally diverse ligands, including vascular endothelial growth factor (VEGF) (Soker et al., 1998) and semaphorins (He and Tessier-Lavigne, 1997; Kolodkin et al., 1997), and plays an essential role in developmental and pathological angiogenesis, arteriogenesis, and vascular permeability (Kitsukawa et al., 1995; Kitsukawa et al., 1997; Soker et al., 1998; Kawasaki et al., 1999; Roth et al., 2016). Our recent studies showed that NRP1 mediates interferon-γ-induced chemokine expression in brain microvascular endothelial cells (Wang et al., 2016), implicating NRP1 in the inflammatory response of endothelial cells. Previous studies reported contrasting effects of TNFα stimulation at 24 h on the expression of NRP1 in endothelial cells (Giraudo et al., 1998; Yang et al., 2004). Therefore, the relevance of TNF for NRP1 expression is not fully clear and the molecular details of the crosstalk between NRP1 and TNFα remain unclear.

In this study, we define the role of NRP1 in the inflammatory response induced by TNFα in vascular endothelial cells. We found that knockdown of NRP1 attenuates TNFα-induced expression of adhesion molecules, leukocyte adhesion and activation of MAPK p38 in endothelial cells. NRP1 co-localized with TNFRs in the same protein complex but did not affect the expression levels of TNFRs and their binding affinities to TNFα. Furthermore, we defined a time-dependent regulation of NRP1 expression by TNFα in endothelial cells. Collectively, our results reveal a novel reciprocal crosstalk between NRP1 and TNFα which contribute to the inflammatory response of endothelial cells.

## Materials and methods

### Cells and reagents

HUVECs (Lonza) were purchased and cultured in EBM medium with the EGM-MV Bulletkit (Lonza) and authenticated by expression of CD31/105, von Williebrand Factor VIII, and positive uptake of acetylated low-density lipoprotein by the manufacture. THP-1 cells were purchased from ATCC (Manassas, VA, United States) and cultured in RPMI 1640 (Gibco) with 10% FBS supplemented with penicillin and streptomycin. No cell lines listed in the database of misidentified cell lines maintained by ICLAC was used in this study. All the cells were negative for *mycoplasma*. shRNA for human NRP1 and controls were from Open Biosystems (Huntsville, AL). The NRP1 shRNA targeting sequence was CCC​TGT​TGG​TTT​CAT​TTG​AAT​A. The NRP1 shRNA-2# targeting sequence was TAA​GAA​TGA​GGA​TAA​CCA​G. Lentivirus for NRP1 shRNA and control shRNA was prepared in 293T cells, which were transfected with targeted gene (pGIPZ-NRP1 shRNA and pGIPZ-control shRNA), pGag.Pol, and pVSV-G (encoding the cDNAs of the proteins that are required for virus packing) as previously described (Wang et al., 2003; Cao et al., 2008). After infection, 2 μg/mL of puromycin was added to the medium for antibiotic selection for 48 h and then cells were used for further experiments. Retrovirus of NRP1 and LacZ were prepared and used as previously described (Wang et al., 2003). Dynasore hydrate was purchased from Sigma-Aldrich and SB203580 were purchased from Tocris Bioscience.

Recombinant TNFα (Final concentration: 5 ng/mL), antibodies against NRP1 (#3725), TNFR1 (#3736), NFκB p65 (#4764) and its phosphorylated form (#3033), IκBα (#4814), p38 MAPK (#9212), phosphorylated p38 MAPK (Thr180/Tyr182, #9215), ERK1/2 (#9102), phosphorylated ERK1/2 (Thr202/Tyr204, #9101), AKT (#4691) and phosphorylated AKT (Ser473, #4060), JNK (#9258) and phosphorylated JNK (Thr183/Tyr185, #4668), and EEA1 (#2411) were purchased from Cell Signaling Technology, Inc. (Danvers, MA). β-Actin antibody (A2228) was purchased from Sigma-Aldrich (St. Louis, MO). TNFR2 antibody (19272-1-AP, Proteintech), TNFR1 antibody (AF225-SP, R&D) and NRP1 antibody (AF3870, R&D) were purchased from R&D and used for immunofluorescent staining. Neutralizing antibodies of TNFR1, TNFR2 and control IgG were from R&D.

### qPCR, western blotting, immunoprecipitation, and immunofluorescent staining

Total mRNA was isolated from cells and tissues using the RNeasy Mini Kit (Qiagen, Hilden, Germany) and was reverse-transcribed by using oligo (dT) priming using the iScript cDNA Synthesis kit (Bio-Rad). qPCR analyses were performed using the ABI 7500 Real-Time PCR System (Applied Biosystems, Foster City, CA) and SYBR Green PCR Master Mix (Applied Biosystems, Warrington, United Kingdom). The results were normalized to the β-actin gene (human). These are the primer sequences: NRP1 forward: 5′-GAC​TGG​GGC​TCA​GAA​TGG-3′, NRP1 reverse: 5′-CTA​TGA​CCG​TGG​GCT​TTT​CT-3′, VCAM1 forward: 5′-ATG ACA TGC TTG AGC CAG G-3′, VCAM1 reverse: 5′-GTG TCT CCT TCT TTG ACA CT-3′, ICAM1 forward: 5′-GCT​GAC​GTG​TGC​AGT​AAT​ACT​GG-3′, ICAM1 reverse: 5′-TTC​TGA​GAC​CTC​TGG​CTT​CGT-3′, β-actin forward: 5′-CCA​ACC​GCG​AGA​AGA​TGA-3′, β-actin reverse: 5′-CCA​GAG​GCG​TAC​AGG​ATA​G-3’.

For total protein lysate extraction, HUVECs were lysed in RIPA buffer supplemented with proteinase and phosphatase inhibitors. For immunoprecipitation, cells were lysed with lysis buffer (HEPES 50 mM, NaCl 125 mM, CHAPS 0.5%, EDTA 1 Mm) supplemented with DTT (2 mM), PMSF (0.2 mM) and proteinase inhibitors. Cell lysates were incubated with primary antibodies conjugated with Magnetic Dynabeads Protein G (#1003D, ThermoFisher) over night.

For Western blotting, equal amounts of protein were loaded onto SDS-PAGE and transferred onto PVDF membranes. Membranes were incubated with specific antibodies at 4°C overnight, and then with HRP-conjugated secondary antibodies (Santa Cruz technology Inc., Dallas, Texas) for 1 h at room temperature. Immunodetection was performed with the Clarity™ Western ECL Blotting Substrates Substrate (Biorad, Hercules, CA) using either X-ray films or a ChemiDoc imaging system (Biorad, Hercules, CA).

For immunofluorescent staining, cells were fixed in pre-warmed 4% paraformaldehyde for 10 min, blocked in 3% BSA for 30 min at room temperature and then incubated with primary antibodies overnight at 4°C. Then cells were washed with PBS 3 times for 5 min, incubated with Alexa Fluor-labeled secondary antibodies (Invitrogen, Eugene, Oregon) at room temperature for 1 h, rinsed with PBS 5 min for 3 times and then mounted in DAPI-containing aqueous mounting media (Vector Laboratories Inc., Burlingame, CA). Images were taken using a Zeiss LSM880 confocal microscopy (Carl Zeiss AG, Oberkochen, Germany).

### EC-leukocyte adhesion assay

THP-1 cells comprise a human acute monocytic leukemia cell line and were used in this study as a model of peripheral blood monocytes. The adhesion assay was performed as previously described with minor modification (Fan et al., 2008). THP-1 cells were labeled with CellTracker™ Orange CMTMR Dye (#C2927, Thermo Fisher Scientific), which was diluted in RPMI 1640 culture medium at a final concentration of 10 μM, for 20 min at 37°C and then THP-1 cells (1.5*10^5^/mL) were co-cultured with control or TNFα-stimulated (5 ng/mL, 20 h) HUVECs in a 24-well plate for 30 min at 37°C. Unbound THP-1 cells were removed by gently rinsing with warm culture medium for three times. Fresh medium was added, and images were acquired with an EVOS cell imaging system (Life Technologies). Four to six images at ×10 magnification were acquired per well and adhered THP-1 cells were manually counted. The experiments were performed in triplicates each time and independently repeated for 3 times.

### Binding assay of GpL-TNFα fusion protein to receptors in HUVECs

The binding assay was performed as previously described with minor modifications (Lang et al., 2016). The recombinant GpL-TNFα and FLAG-TNFα were cloned and produced as previously described (Lang et al., 2016). Control and NRP1 knockdown HUVECs were counted and plated in a 24-well plate (5*10^4^/well), and the binding assay was performed 2 h later to minimize the interference of potentially different cell proliferation between two groups. To determine the non-specific binding, half of the wells were pre-incubated with an excess of conventional FLAG-TNFα (5 μg/mL) for 1 h at 37°C, then all the wells were incubated with the indicated concentrations of GpL-TNFα for 30 min at 37°C. Unbound GpL-TNFα was removed by 3 rapid washes in ice-cold PBS and 3 rapid washes in room-temperature PBS, and cells were scratched with a mini cell scraper in 100 μL PBS. The GpL activity was measured in a 96-well plate using a Spectramax i3x system (Molecular Devices, Sunnyvale, CA) with one injector loaded with Gaussia luciferase substrates (Gaussia luciferase kit, New England Biolabs, Ipswich, MA).

### Binding assay of NRP1-Fc-GpL and GpL-TNC-TNFα fusion proteins to plastic immobilized TNFR2-Fc

TNFR2-Fc was used for determining total binding and TRAILR1-Fc was for the determination of unspecific binding. They were solved in coating buffer (2 μg/mL) and immobilized at 4°C overnight on black 96-well plastic plates. After 3 washes with PBST, wells were blocked with 10% FCS in PBS for 1 h at room temperature. After additional three washes with PBST, the wells were incubated pairwise for 2 h at 37 with the indicated concentrations of GpL-TNC-TNF and NRP1-Fc-GpL. After removal of unbound molecules by 10 washes with ice-cold PBS, wells were filled with 50 µL/well 0.5% FCS in RPMI 1640 medium. GpL activities were determined after adding 25 µL/well GpL substrate (1.5 μM coelenterazine in PBS) and measured with LUmo luminometer (anthos Mikrosysteme GmbH, Frieoythe, Germany). Specific binding-values were calculated by subtracting the unspecific binding-values derived of the TRAILR1-Fc coated wells from the total binding-values derived of the corresponding TNFR2-Fc coated wells. Specific binding-values of GpL-TNC-TNF were fitted to a one-side specific binding plot by non-linear regression in GaphPad Prism 5 Software (San Diego, CA, United States) to derive maximal binding (Bmax). To average the results of independent experiments specific binding-values of each experiment were normalized against the Bmax-value derived of the corresponding GpL-TNC-TNF specific binding curve. Specific binding-values in % of Bmax from the independent blots were then averaged and analyzed again by non-linear regression to determine the KD-value from the averaged data.

### Binding assay of NRP1-Fc-GpL and GpL-TNC-TNFα fusion proteins to cells expressing GPI-anchored TNFRs

HEK293 cells were transiently transfected with expression plasmids encoding the GPI-anchored ectodomains of TNFR1 and TNFR2 or with empty vector (EV) using polyethylenimine (PEI). The expression vectors encoding fusion proteins of the TNFR1 and TNFR2 ectodomain with a GPI-anchoring tag are a kind gift of Prof. Pascal Schneider (University of Epalinges, Switzerland) and are described elsewhere in details (Bossen et al., 2006). After 2 days, receptor transfectants and empty vector transfected cells were harvested and aliquoted (100 µL with 5 × 10^5^ cell) in RPMI medium, 10% FCS. Receptor- and EV-transfected cells were then incubated pairwise for 2 h at 37 celsius with the indicated concentrations of GpL-TNC-TNF and NRP1-Fc-GpL. After removal of unbound molecules by 3 washes with 1 mL ice-cold PBS, cells were resuspended in 50 µL 0.5% FCS in RPMI medium and transferred to black 96-well plates to measure GpL activity as described above. Specific binding-values were obtained by subtracting the unspecific binding-values derived of the EV transfected cells from the total binding-values derived of the TNFR1-and TNFR2-GPI expressing transfectants. Specific binding of GpL-TNC-TNF were again fitted to a one-side specific binding plot by non-linear regression (GaphPad Prism 5 Software). The resulting Bmax-value was again used to normalize the specific binding-values. Finally, the normalized specific binding-values of each experiment were averaged and reanalyzed by non-linear regression to determine the KD-value from the averaged data.

To analyze the effect of soluble TNF on the interaction of NRP1-Fc-GpL and GpL-TNC-TNFα with TNFR1-GPI and TNFR2-GPI, aliquots with the corresponding receptor transfectants were incubated with 100 ng/mL of GpL-TNC-TNF and NRP1-Fc-GpL in the absence and presence of 200 ng/mL TNF. After removal of unbound molecules (3 washes, 1 mL ice-cold PBS) cells were resuspended in 50 µL 0.5 FCS in RPMI medium and transferred to black 96-well plates to measure GpL activity.

### Molecular docking

The docking analysis was performed as previously described (Coban et al., 2021). The proteins sequences were used to generate a complete structural model, and were constructed based on multiple alignments, in which each domain was modeled as a separate unit, which were then assembled into a final composite hybrid model. The model was generated from consensus between the programs PHYRE (Kelley et al., 2015), PRIME (Prime v3.0, Schrodinger, LLC, New York, NY) (Jacobson et al., 2002; Jacobson et al., 2004), and YASARA SSP/Homology/PSSM Method (Hooft et al., 1996a; Hooft et al., 1996b; King and Sternberg, 1996; Qiu and Elber, 2006). The variable loops and gaps were filled using homology and knowledge-based potentials with YASARA. Each missing loop was modeled using the Loop Search module in Sybyl 8.0 or with YASARA loop modeler (Hooft et al., 1996a; Hooft et al., 1996b; King and Sternberg, 1996; Muckstein et al., 2002; Canutescu et al., 2003; Qiu and Elber, 2006; Krieger et al., 2009). The selection of final loops was based on highest homology, as well as lowest root mean square deviations (RMSDs). The side chains and rotamers were adjusted with empirical potentials, simulated annealing with explicit solvent, and small equilibration simulations using YASARA’s refinement protocol. These were verified by WHAT-IF and PROCHECK (Hodsdon et al., 1996). Refinement of the hybrid model was carried out using a limited molecular dynamics-based refinement in YASARA consisting of the Secondary Structure Prediction (SSP) for YASARA parameterization, pKa assignment, solvation and simulated annealing and pre-equilibration setup, and energy minimizations. Both homology and fold recognition were considered, and a final refinement with the entire model was completed using YASARA for 250 ps of MD embedded in an empirical force field. The model was then subjected to energy optimization with Polak-Ribiere conjugate gradient (PRCG) with an R-dependent dielectric for finalization. Subsequent relaxation MD simulations of long length (10–100X longer) were completed to get complexes with optimal fit and inter-/intra-molecular arrangement for interrogation of protein-protein and protein draggability assessments.

### Statistics

All analysis were performed with GraphPad Prism 5 (GraphPad Software Inc., La Jolla, CA, United States). Experiments were routinely repeated at least three times, and the number was increased according to effect size or sample variation. Values were expressed as mean ± SD. Statistical differences were determined to be significant at *p* < 0.05. For comparison between two groups, an unpaired two-tailed Student's t-test was performed. For comparison among three or more groups, one-way ANOVA followed by Tukey test was used.

## Results

### Knockdown of NRP1 attenuates TNFα-induced inflammatory responses in endothelial cells

**FIGURE 1:**
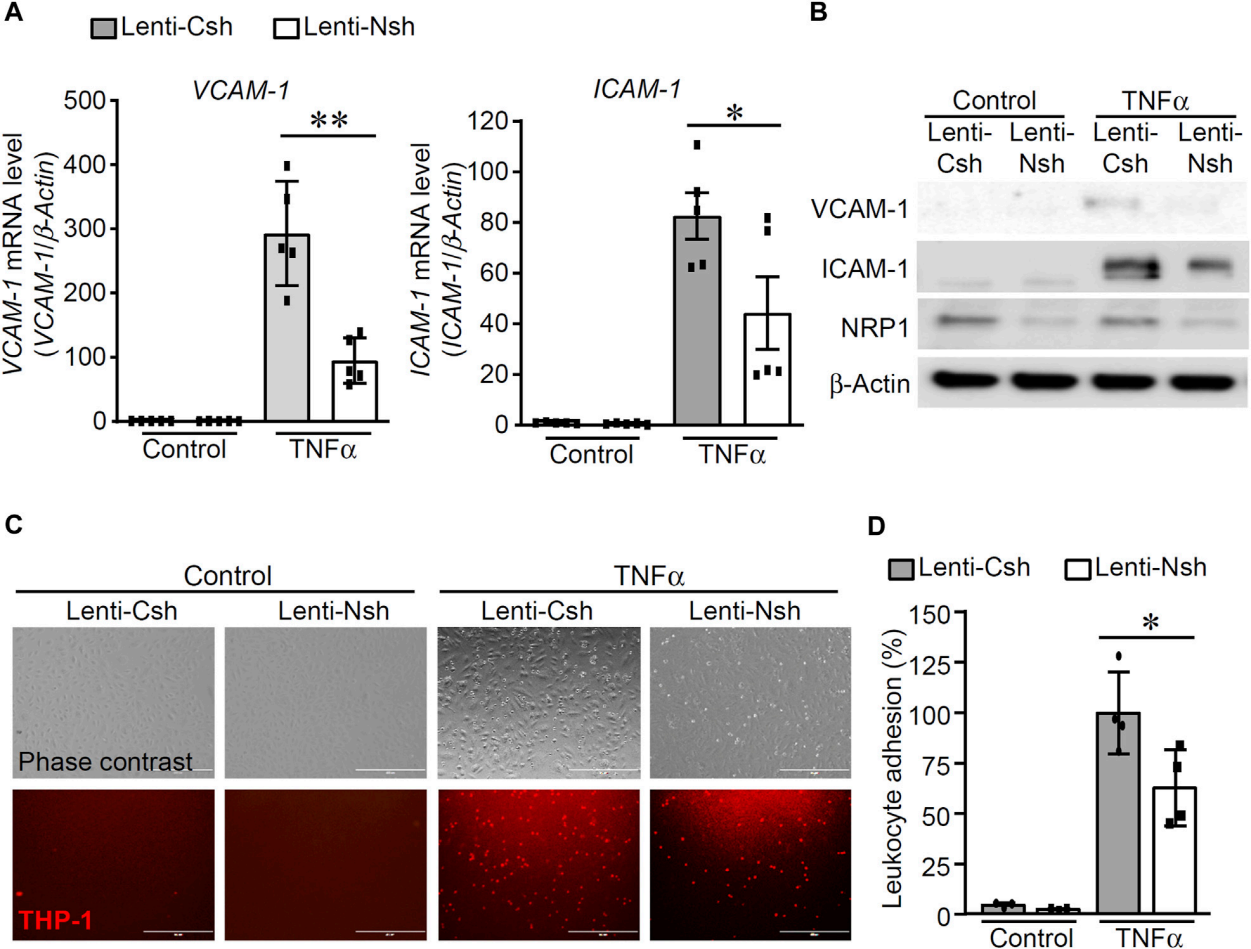
Knockdown of NRP1 attenuated TNFα-induced expression of adhesion molecules in endothelial cells. **(A, B)** Control and NRP1 knockdown Human Umbilical Vein Endothelial Cells (HUVECs) were stimulated with recombinant human TNFα (5 ng/mL) for 20 h and then subjected to qPCR **(A)** and Western blotting **(B)**. N = 3-5 per group in Fig.1A and the results in Fig.1B are represented from three independent experiments. **(C,D)** Control and NRP1 knockdown HUVECs at 80% confluency were stimulated with recombinant human TNFα (5 ng/mL) for 20 h, then co-cultured with CMTMR-labeled THP-1 cells for 30 min. Unbounded THP-1 cells were removed by gently rinsing with warm culture medium. Images were acquired using an EVOS cell imaging system **(C)**. Representative images are from four to six images per group. The adhered THP-1 cells were manually counted and analyzed **(D)**. The experiments were performed in triplicates and independently repeated for 3 times. Scale bar, 400 μm *, *p* < 0.05, **, *p* < 0.01, in unpaired T-test.

To examine the effect of NRP1 on TNFα-induced endothelial cell inflammatory responses, HUVECs were infected with lentivirus expressing control shRNA (Lenti-Csh) or NRP1 shRNA (Lenti-Nsh), selected with puromycin, and then stimulated with TNFα. As shown in Figure 1A, B, knockdown of NRP1 attenuated TNFα-induced expression of the adhesion molecules ICAM-1 and VCAM-1, both at the mRNA and protein levels.

As adhesion molecules mediate the rolling and association of leukocytes onto endothelial cells, leukocyte adhesion assays were performed to examine whether knockdown of NRP1 inhibits the adhesion of leukocyte THP-1 cells to ECs upon TNFα stimulation. Cell-tracker labeled THP-1 cells were co-cultured with control and NRP1 knockdown HUVECs, which were stimulated with control or TNFα for 20 h, respectively. As shown in Figure 1C, D, THP-1 observed adhesion was minimal in both control shRNA and NRP1 shRNA groups under basal conditions. Upon TNFα stimulation, significantly increased THP-1 adhesion was observed. NRP1 knockdown resulted in significantly decreased leukocyte adhesion (90.8 ± 9.1 cells/field in the control shRNA group *versus* 57.0 ± 8.6 cells/field in the NRP1 knockdown group, *p* < 0.05). HUVECs were also infected with lentivirus expressing an independent NRP1 shRNA to confirm that knockdown of NRP1 in HUVECs reduced the leukocyte adhesion upon TNFα stimulation (114.5 ± 14.5 cells/field in the control group *versus* 55.7 ± 11.0 cells/field in the NRP1 knockdown group, *p* < 0.05, Supplementary Figure S1). These results suggested that NRP1 is directly involved in the regulation of TNFα-induced inflammatory responses in endothelial cells.

### Knockdown of NRP1 decreases TNFα-stimulated phosphorylation of p38 MAPK

**FIGURE 2:**
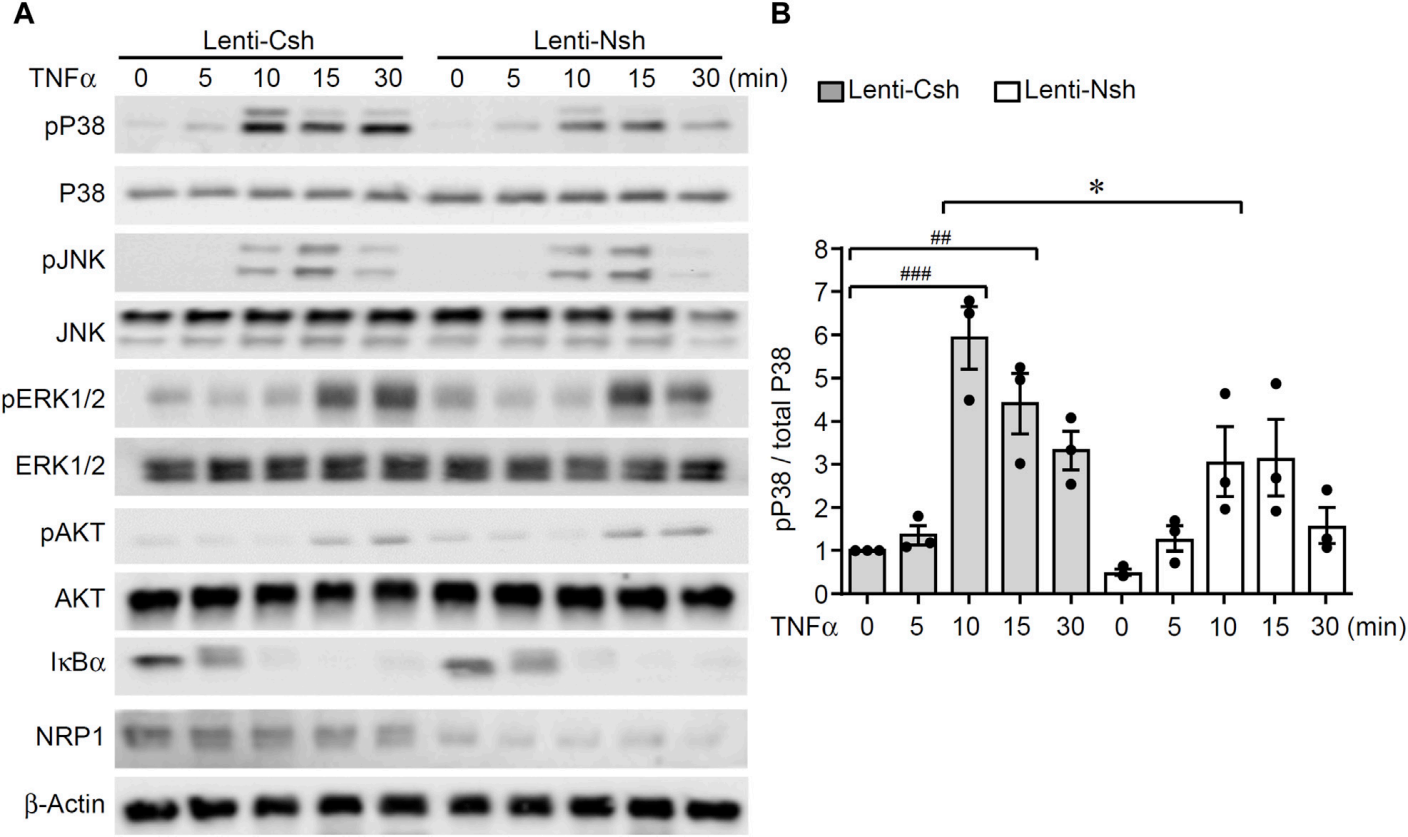
Knockdown of NRP1 reduced TNFα-stimulated phosphorylation of p38 MAPK in endothelial cells. **(A, B)** HUVECs were infected with lentivirus expressing control shRNA or NRP1 shRNA, selected with puromycin for 48 h, and then stimulated with TNFα (5 ng/mL) for the indicated times, protein lysates were collected and subjected to Western blotting. Results are representative of 3 independent experiments. Densitometry of the immunoblots of p38 MAPK was performed using ImageJ software **(B)**. *, *p* < 0.05, ##, *p* < 0.01, ###, *p* < 0.001 in One-way ANOVA test.

To understand the molecular mechanisms of the enhancing effect of NRP1 on TNF signaling in endothelial cells, we examined the activation of TNFα-stimulated signaling pathways. As shown in Figure 2A, B; Supplementary Figure S2, TNFα induced the degradation of IκBα and phosphorylation of p38 MAPK, JNK, ERK1/2 and AKT in endothelial cells. Compared with the control group, TNFα-induced phosphorylation of p38 MAPK 10–30 min post stimulation was reduced while the other signaling pathways remained unaffected. This analysis suggests that knockdown of NRP1 selectively suppressed TNF-induced activation of p38 MAPK in endothelial cells. To examine the relevance of p38 MAPK for the reduced inflammatory response of NRP1 knockdown cells, the inhibitor SB203580 was administered prior to TNFα stimulation. SB203580 further reduced the mRNA expression of VCAM1, E-selectin, but not ICAM-1 in NRP1 knockdown cells (Supplementary Figure S3), suggesting that activation of MAPK p38 is partially involved in the regulation of adhesion molecules in NRP1 knockdown endothelial cells.

### NRP1 and TNFR1 are in the same protein complex in endothelial cells

**FIGURE 3:**
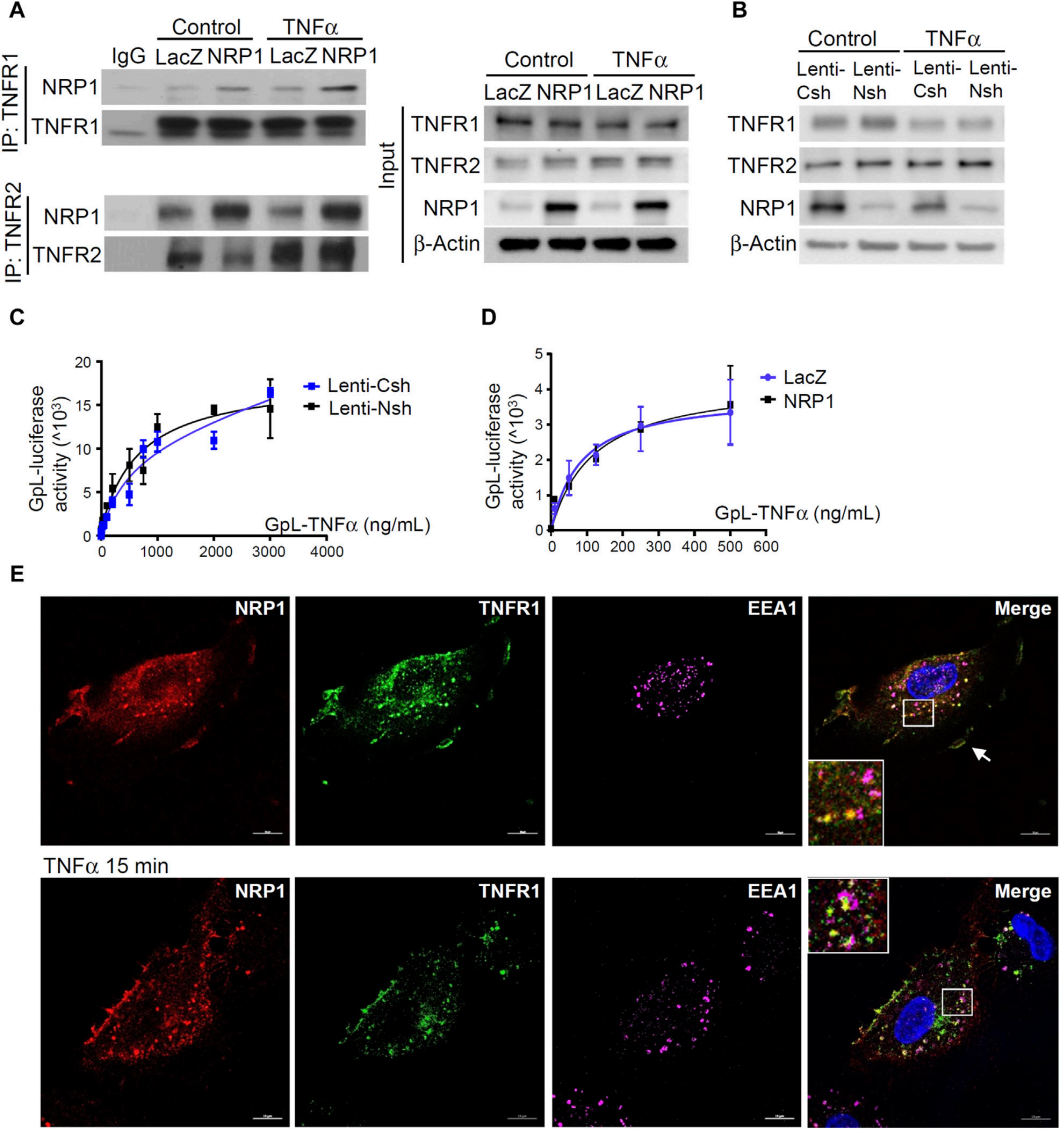
NRP1 and TNFRs are in the same protein complexes. **(A)** HUVECs were infected with retrovirus expressing NRP1 or LacZ as a control, and then stimulated with TNFα (5 ng/mL, 15 min). Cell lysates were immunoprecipated with anti-TNFR1 or TNFR2 antibodies and immunoprecipates were analyzed for the presence of the TNFRs and NRP1 by Western blotting (left panels). Cell lysates were immunoblotted as load control (right panel). **(B)** HUVECs were infected lentivirus expressing control-shRNA and NRP1-shRNA, respectively, selected with puromycin for 48 h, stimulated with TNFα and then subjected to Western blotting. **(C, D)** HUVECs (5*10^4^/well) were plated into a 24-well plate. Half of the wells were pre-incubated with an excess of “non-GpL” TNFα (2 μg/mL) to determine the non-specific binding of *Gaussia. princeps* luciferase TNFα (GpL-TNFα). Then all the wells were incubated with GpL-TNFα at different concentrations for 30 min at 37°C. The non-specific binding values of the “non-GpL” TNFα groups were subtracted from the corresponding total binding values. The data were analyzed by a non-linear regression to a single site using the GraphPad Prism 5 software. The experiments were performed in duplicates and independently repeated for 3 times. **(E)** HUVECs were stimulated with TNFα (5 ng/mL) for 15 min and then subjected to immunofluorescent staining with the indicated antibodies. Nuclei were counterstained with DAPI. Co-localization of NRP1 and TNFR1 in both plasmal membrane (arrow) and cytoplasm were observed. Scale bar, 10 μm.

TNFα binds to its two cognate membrane receptors TNFR1 and TNFR2 to activate downstream signaling pathways. Given that NRP1 is predominantly localized in the cell membrane and acts as a co-receptor for many receptors, we explored the possibility of a direct or indirect interaction between NRP1 and the two TNFRs. As shown in Figure 3A, The NRP1-TNFR1 and NRP1-TNFR2 associations were confirmed by immunoprecipitation experiments with polyclonal anti-TNFR1 and anti-TNFR2 antibodies, respectively. NRP1 coimmunoprecipitated with TNFR1 as well as TNFR2 both in HUVEC with endogenous NRP1 expression and HUVECs cells stably expressing increased levels of NRP1. The association of NRP1 with TNFR1 and TNFR2 was largely independent from TNF (Figure 3A; Supplementary Figure S4).

Notably, Western blotting and cellular binding studies with HUVEC cells and GpL-TNFα(24), a TNF fusion protein with *Gaussia princeps* luciferase as an easily quantifiable reporter domain, showed that neither overexpression nor knockdown of NRP1 affected the cell surface accessible levels of the two TNFRs nor their affinity for TNFα (Figure 3B–D). Thus, NRP1 is not functioning as a primary receptor for TNFα.

Endocytosis and intracellular trafficking play critical roles in the degradation, recycling, and signal transduction of TNFRs (Schneider-Brachert et al., 2004; Choi et al., 2014). Immunofluorescent staining was performed to examine the co-localization of NRP1, TNFR1 and early endosome antigen 1 (EEA1), a marker of early endosome, which serve as sorting stations for the degradation and recycling of internalized membrane receptors. The co-localization of NRP1 with TNFR1 was predominantly in cytoplasm of HUVECs (Figure 3E). In contrast, there was no prominent co-localization of NRP1 and TNFR2 (Supplementary Figure S4C). Importantly, upon TNFα stimulation for 15 min, more NRP1 and TNFR1 co-localized with EEA1, suggesting that NRP1 is involved in the intracellular trafficking of TNFR1. To further define the role of intracellular trafficking for the reduced expression of adhesion molecules in NRP1 knockdown cells, control and NRP1 knockdown HUVECs were pre-treated with dynasore, an inhibitor of internalization of membrane receptors. Interestingly, in the presence of dynasore, the mRNA expression of ICAM-1, but not VCAM-1 or E-selectin, was reversed in NRP1 knockdown HUVECs (Supplementary Figure S5), suggesting that the anti-inflammatory effect of NRP1 knockdown partially relies on endocytosis.

### NRP1 does not affect TNF-TNFR interaction or directly bind to TNFα/TNFRs

**FIGURE 4:**
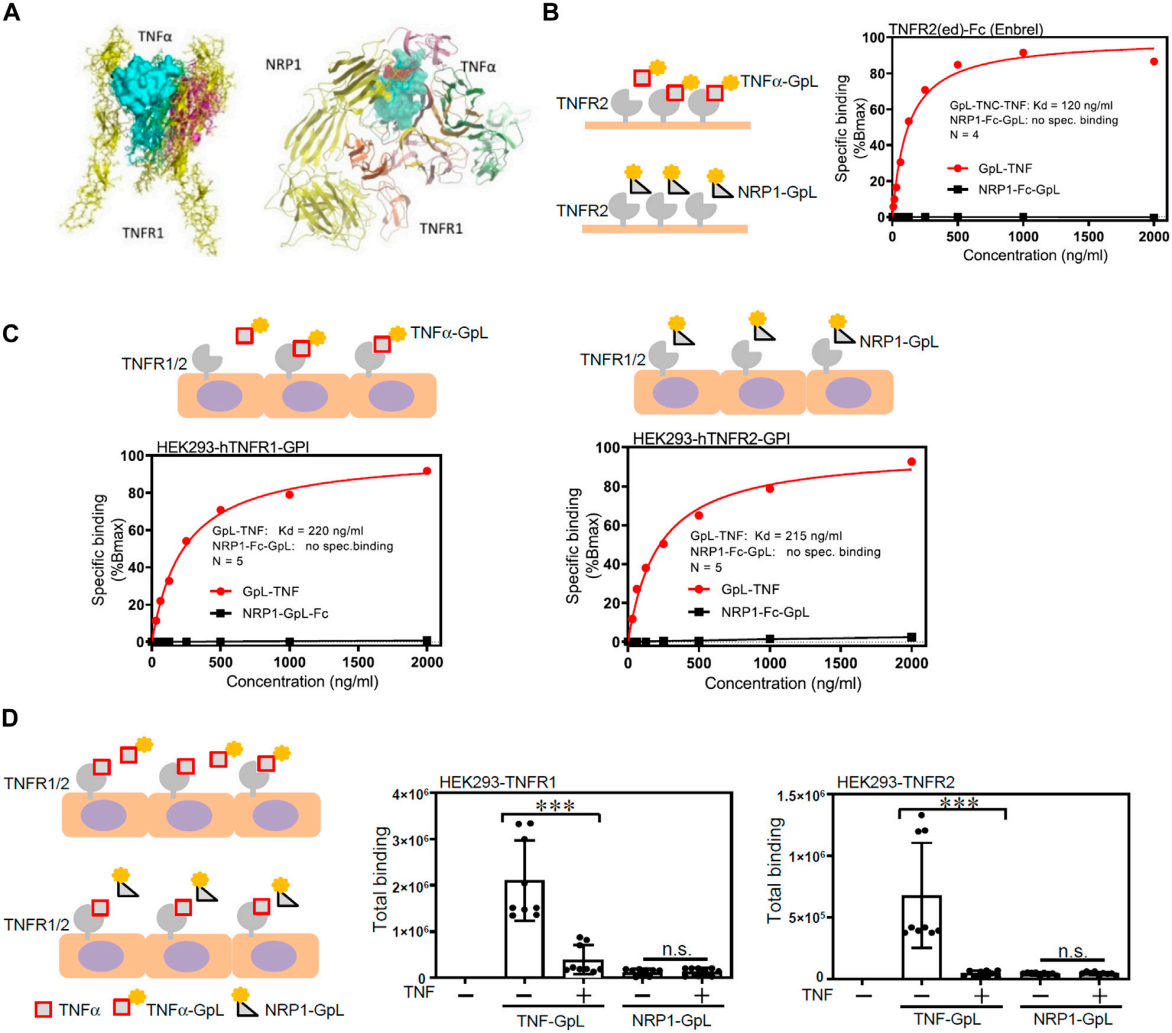
NRP1 does not directly bind to TNFα/TNFRs. **(A)** Molecular docking model of NRP1, TNFα and TNFR1. **(B)** Specific binding of GpL-TNC-TNF (TNFα-GpL) and NRP1-Fc-GpL (NRP1-GpL) to plastic immobilized TNFR2-Fc. Data shown have been averaged from four independent experiments, in which specific binding values have been normalized against the calculated Bmax of TNFα-GpL binding. **(C)** Specific binding of TNFα-GpL and NRP1-GpL to HEK293 cells transiently expressing the GPI-anchored ectodomains of TNFR1 (left panel) and TNFR2 (right panel). Data shown have been averaged from five independent experiments, in which specific binding values have been normalized against the calculated Bmax of TNFα-GpL binding. **(D)** HEK293 cells transiently expressing the GPI-anchored ectodomains of TNFR1 and TNFR2 were incubated with 100 ng/mL of TNFα-GpL and NRP1-GpL in the absence and presence of 200 ng/mL TNF. Total binding values after 2 h at 37°C are shown and were derived from three independent experiments. The TNFα-GpL and NRP1-GpL total binding-values plus minus TNF were analyzed in each case by two-tailed T-test. ***, *p* < 0.001; n. s., not significant, in unpaired T-test.

To further examine the molecular details of the crosstalk between NRP1 and TNFR, the potential for NRP1 to couple as a co-receptor to the TNF/TNFR complexes was assessed using a computational protein docking model. Based on the crystal structures, a potential binding area was assigned for docking of NRP1 in the presence of TNFR1. The greatest scored docking pose showed three domains that could mediate contacts between NRP1 and TNFR1 (Figure 4A). To examine a possible direct interaction between NRP1 and the TNFRs, we evaluated the binding of an NRP1-Fc-GpL fusion protein to microplate wells coated with TNFα and TNFR2-Fc. We did not observe an increased binding of NRP1-Fc-GpL to TNFα and TNFR2-Fc while an efficient binding between TNFα and TNFR2 was observed (Figure 4B; Supplementary Figure S6A). We also performed cellular binding studies with cells transiently expressing constructs encoding GPI-anchored variants of TNFR1 and TNFR2. While the GpL-TNF fusion protein showed again strong dose dependent binding, there was no evidence for binding of NRP1-Fc-GpL (Figure 4C). We also analyzed binding between TNFR1-and TNFR2-GPI and NRP1-Fc-GpL in the presence of TNF. Again, there was no evidence for a direct TNFR-NRP1 interaction (Figure 4D). Furthermore, to validate the binding of TNFα to cell surface NRP1 which is in an oligomeric state (Chen et al., 1998; Nakamura et al., 1998; Takahashi et al., 1998; Wajant et al., 2003; Kalliolias and Ivashkiv, 2016), HEK293 cells were transfected with plasmids to overexpress TNFR1, TNFR2 and NRP1, respectively, and then co-cultured with GpL-TNFα. While TNFR1-and TNFR-2 overexpressed HEK293 cells exhibited extensive increase of luciferase activity, indicatives of binding of GpL-TNFα, NRP1 overexpressed cells showed limited increase of luciferase activity (Supplementary Figure S6B).

### TNFα decreases NRP1 expression in endothelial cells through TNFR1-NFκB dependent mechanism

**FIGURE 5:**
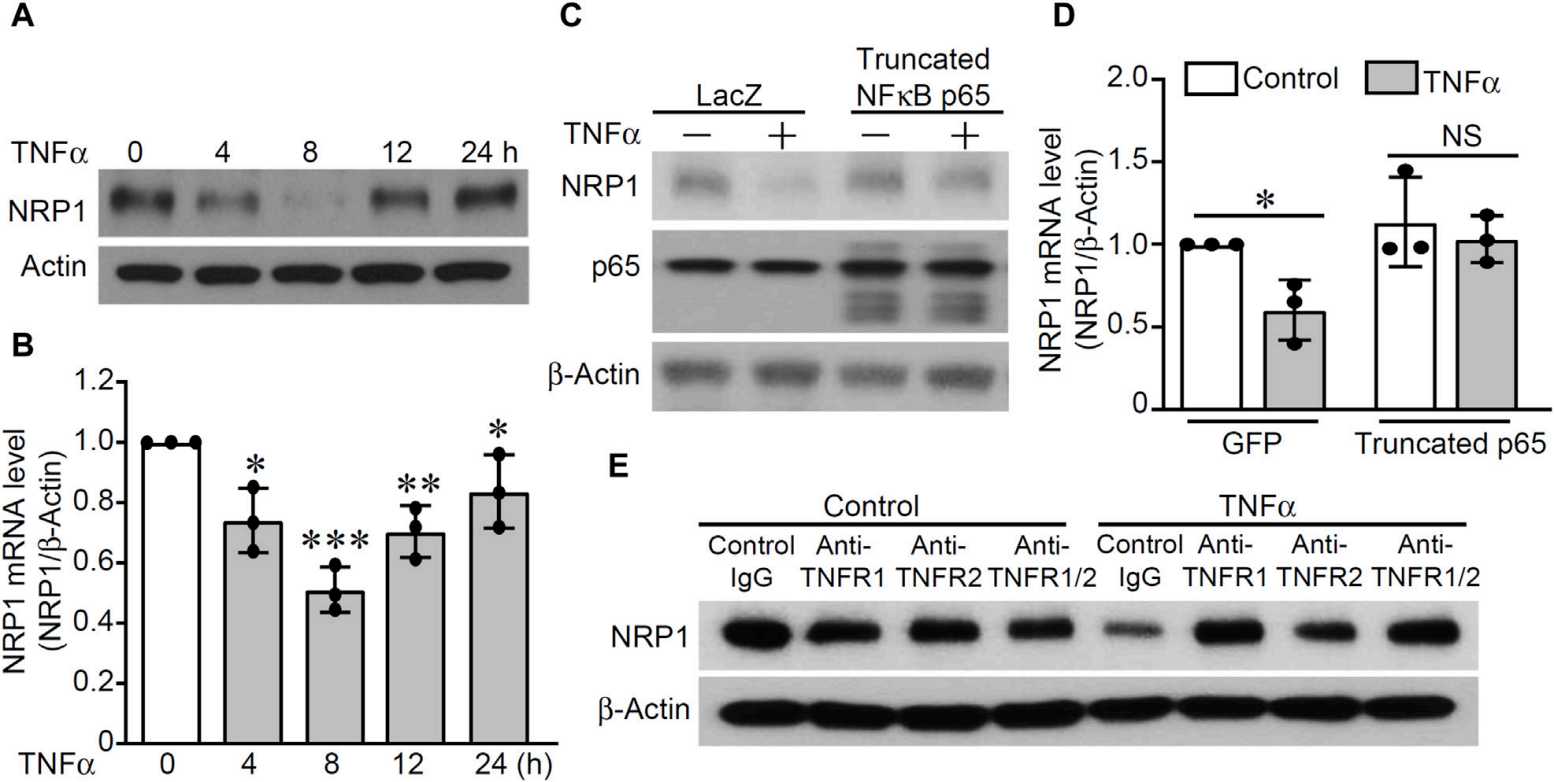
TNFα downregulates NRP1 in a TNFR1-NFκB dependent mechanism. **(A, B)** HUVECs were stimulated with TNFα (5 ng/mL) at the indicated time points and then subjected to Western blotting **(A)** and qRT-PCR. **(C, D)** HUVECs were infected with adenovirus mutant p65 or adenovirus GFP as control and then treated with TNFα for 8 h. Expression of NRP-1, p65, and β-actin were analyzed with Western blotting **(C)** and qRT-PCR **(D)**. **(E)** HUVECs were pretreated with TNFR1 neutralizing antibody, TNFR2 neutralizing antibody or Isotype control for 1 h and then stimulated with TNFα for 8 h. Total protein was harvested and subjected to Western blotting. *, *p* < 0.05, **, *p* < 0.01, ***, *p* < 0.001, NS, not significant, in one-way ANOVA followed by Tukey test **(B)** or T-test **(D)**.

Previous studies described potential regulation of TNFα on NRP1, however, higher dose of TNFα at 20 ng/mL or longer time point of 24 h were used (Giraudo et al., 1998; Yang et al., 2004). To examine the effect of TNFα on NRP1 expression, HUVECs were stimulated with TNFα (5 ng/mL) and the mRNA and protein levels of NRP1 were analyzed at different time points. As shown in Figure 5A, B and Supplementary Figure S7, both NRP1 mRNA and protein levels were decreased after a 4 h treatment with TNFα, continued to decline at 8 h, began increasing at 12 h, and had nearly returned to baseline levels after 24 h (Figure 5A&B). Adenovirus-mediated overexpression of a truncated form of NFκB p65, which lacked the transactivation domain and significantly blocked TNFα-induced NFκB transactivation activity in an NFκB reporter assay (Supplementary Figure S8), rescued NRP1 from TNFα-induced downregulation (Figure 5C, D). Furthermore, neutralizing antibodies against TNFR1 efficiently inhibited TNFα-induced suppression of NRP1 while blocking TNFR2 exhibited a little effect (Figure 5E). The combination of TNFR1 and TNFR2 neutralizing antibodies did not further potentiate the effect of TNFR1 neutralizing antibody, suggesting that predominantly TNFR1 mediates the effect of TNFα on NRP1 expression. Taken together, these results suggest that TNFα downregulates NRP1 expression in a TNFR1-NFκB dependent manner.

## Discussion

In this study we studied the role of NRP1, a co-receptor of several structurally diverse ligands, in TNFα-induced inflammatory responses in endothelial cells. A crucial role of NRP1 in the TNFα-induced endothelial inflammatory responses was evident from decreased leukocyte adhesion and reduced expression of adhesion molecules, including ICAM-1 and VCAM-1, upon NRP1 knockdown in endothelial cells (Figure 1). Notably, TNFα induced two species of ICAM-1 molecules with two different molecular weights, which likely reflect different glycosylation forms of ICAM-1 (Scott et al., 2013), and both forms were reduced by NRP1 knockdown. As the recruitment of leukocytes to endothelium is an initial stage in inflammatory diseases (Steyers and Miller, 2014), including atherosclerosis (Gimbrone and Garcia-Cardena, 2016), our results suggest that NRP1 could potentially be modulated to maintain vascular health. In line with the previous studies of NRP1 in cultured dendritic cells and a mouse chronic bladder inflammation model (Grage-Griebenow et al., 2007; Saban et al., 2010), the role of NRP1 in the regulation of inflammation is also consistent with our previous study demonstrating knockdown of NRP1 in endothelial cells reduced inflammation in a murine experimental allergic encephalomyelitis model (Wang et al., 2016). Meanwhile, NRP1 was recently shown to have an immune suppressive role in CD4^+^ T cells and regulatory T cells in immune diseases and tumor immunity (Solomon et al., 2011; Kumanogoh and Kikutani, 2013; Overacre-Delgoffe et al., 2017). Interestingly, a recent study demonstrated that reducing endothelial NRP1 increases inflammatory cytokine expression and leukocyte rolling through a mechanism involving shear stress-mediated interaction with vascular endothelial-cadherin (VE-cadherin) and transforming growth factor-β receptor II (Bosseboeuf et al., 2023), suggesting the complex roles of NRP1 in different contexts. Our results provide insights into elucidation of the distinct role of NRP1 in various cell types in the pathogenesis of inflammatory diseases. Notably, endocytosis inhibitor Dynasore was shown to reverse the mRNA levels of ICAM-1 whereas an inhibitor of p38 MAPK further reduced the expression of VCAM-1 and E-selectin in NRP1 knockdown cells (Supplementary Figures S3, S5), supporting the idea that multiple mechanisms including endocytosis and p38 MAPK are involved in the reduced inflammatory response of NRP1 knockdown cells.

Modulation of NRP1 expression did not significantly alter the levels of TNFRs or the binding affinities of TNFRs to TNFRs in these cells (Figure 3), consequently, knockdown of NRP1 did not lead to a universal inhibition on the signaling pathways induced by TNFα (Figure 2). Instead, a specific reduction of p38 MAPK phosphorylation was observed in the NRP1 knockdown HUVECs (Figure 2). NRP1-mediated activation of p38 MAPK was shown previously to determine the divergent response of endothelial cells to different VEGF isoforms, including VEGF165 and VEGF121, both of which stimulate the similar levels of VEGFR2 phosphorylation (Kawamura et al., 2008). However, the mechanism through which NRP1 mediates the phosphorylation of MAPK p38 requires future study. In this study, we examined the activation of these signaling pathways within 30 min after TNFα stimulation. While activation of MAPKs typically returns to baseline within 1 h, oscillation of NFκB activation persists hours after TNFα stimulation (Nelson et al., 2004). Knockdown of NRP1 did not significantly alter the IκBα levels at 30 min (Figure 2) or NFκB reporter luciferase activity 24 h after TNFα stimulation (data not shown). However, given the long-term effect of TNFα on several other pathways, regulating, e.g., epigenetic remodeling, cholesterol synthesis, etc., (Mechtcheriakova et al., 2001; Orr et al., 2005; Fowler et al., 2022), our current data does not exclude the possibilities that NRP1 may affect such pathways in ECs.

The immunoprecipitation data (Figure 3) support that NRP1 and TNFRs are in the same protein complex in HUVECs. Immunoprecipitation data (Figure 3) show that NRP1 and TNFRs are part of a protein complex in HUVECs. TNFR2 displayed a stronger signal in immunoprecipitation but was less prominent in the co-localization assay (Supplementary Figure S4C), likely due to different antibody clones used. Approaches such as quantitative proteomic analysis of NRP1 immunoprecipitates could be used to compare the levels of TNFR1 and TNFR2 in the future studies. Importantly, cellular binding assays do not support a direct interaction of the ectodomains of NRP1 and TNFRs (Figure 4). These data do not exclude the possibility that a third protein/factor is involved and mediates the interaction between NRP1 and TNFRs. It is also possible that the NRP1-TNFR1 interaction requires the transmembrane and/or intracellular domains. Although NRP1 has a short membrane-spanning domain with 23 amino acids and a small cytoplasmic domain with 44 amino acids, its C-terminal SEA motif interacts with the PDZ protein synectin/GIPC1 to mediate protein trafficking (Cai and Reed, 1999). A recent study reveals a new beta hairpin structure in the NRP1 cytosolic tail, which interacts with endosomal SNX-BAR sorting complex promoting exit 1 to facilitate the endosomal trafficking of the SARS-CoV-2 spike protein (Simonetti et al., 2022). Given the co-localization of TNFRs and NRP1 in endothelial cells, our results do not exclude the possibility that NRP1 and TNFRs are part of the same plasma membrane compartment such as lipid rafts and intracellular organelles including endosomes, which were not disrupted efficiently by the immunoprecipitation buffer. Although a binding interface between NRP1 and TNFα/TNFRs was predicated in the protein docking model, the binding assays in cell-free microplates and cultured cells did not support the direct binding of NRP1 to TNFα/TNFRs (Figure 4). Taken together, the current study does not support that NRP1 act as co-receptor of TNFα.

Given the known role of NRP1 in intracellular trafficking and the observed co-localization of NRP1 and TNFRs in both plasmal membrane and cytoplasm, it is likely that NRP1 is involved in the endocytosis and endosomal sorting of TNFRs, a process necessary for modulating the magnitude of TNFα signaling (Choi et al., 2014) and subsequently regulating the expression of adhesion molecules. Indeed, the co-localization of TNFR1 and NRP1 in endosome was observed following TNFα stimulation (Figure 3). A limitation of this study is examining NRP1 and TNFR co-localization only in subconfluent HUVECs. Confluent and subconfluent ECs exhibit significant differences in plasma membrane structure and functions (Corvera et al., 2000), which could potentially alter the co-localization of TNFRs and NRP1. Indeed, a recent study reported that NRP1 forms a complex with VE-cadherin to stabilize adherens junction in ECs (Bosseboeuf et al., 2023). Future studies should investigate how NRP1 coordinates interactions with various cell membrane proteins in different contexts, such as under shear stress. Importantly, the endocytosis inhibitor dynasore reversed the expression of ICAM-1, but not VCAM-1 or E-selectin, in NRP1 knockdown cells (Supplementary Figure S5), indicating the involvement of both endocytosis-dependent and -independent mechanisms in the downregulation of adhesion molecules in NRP1 knockdown cells. Interestingly, retrovirus-mediated overexpression of NRP1 did not show any pro-inflammatory effect or significantly affected TNFα-induced phosphorylation of MAPK p38 (Supplementary Figure S9). It is likely that endogenous NRP1 levels are sufficient to saturate the NRP1-TNFR crosstalk, and additional NRP1 does not enhance this effect. Future studies could investigate the impact of NRP1 overexpression and knockdown under conditions with varying levels of TNFRs. Our results demonstrated that TNFα downregulated the expression NRP1 through a TNFR1-NFκB dependent mechanism in endothelial cells (Figure 5). Previous studies have reported the controversial regulation of long-term stimulation TNFα on NRP1, which is accompanied with the different angiogenetic actions of TNFα (Sainson et al., 2008). It is likely that TNFα dynamically controls the expression of NRP1 to induce pleiotropic signaling pathways in inflammatory response initiation and resolution, and angiogenesis. The doses of TNFα could also potentially affect the crosstalk between NRP1 and TNFRs. Considering that higher doses of TNFα, such as 20 ng/mL, have consistently been shown to induce cytotoxicity in HUVECs (Zhou et al., 2017; Wang et al., 2020), we opted not to investigate the difference between control and NRP1 knockdown cells under TNFα stimulation doses, which is a limitation of the current study. The mutant form of NFκB p65 reversed the levels of NRP1, suggesting that the activation of NFκB p65 represses NRP1 expression. The repressive effect of NFκB p65 is likely mediated by its methylation at lysine 310 (Levy et al., 2011), miRNAs (Lee et al., 2014; Lukiw, 2022) and interactions with other transcriptional factors such as FOXO (Belguise and Sonenshein, 2007; Liu et al., 2008; Hwang et al., 2010) etc., which still need further investigation. In this study, the TNFR1 neutralizing antibody more effectively reversed TNFα-induced NRP1 reduction compared to the TNFR2 antibody. The combination of both antibodies showed no additive effect, suggesting the redundant role of TNFR2 in this context. As a downstream target of both TNFRs, NFκB can be activated by redundant signaling molecules (Wajant and Scheurich, 2011). Additionally, soluble TNFα binds both TNFRs but strongly activates only TNFR1, which may explain the predominant effect of TNFR1 on NRP1 downregulation (Wajant and Siegmund, 2019).

In conclusion, our results suggest that NRP1 is required for TNFα-induced p38 activation and thus the TNFα-stimulated inflammatory response in endothelial cells. TNFα further downregulates the expression of NRP1 through the classical NFκB pathway constituting a potential negative feedback loop to limit p38 signaling. Our results will help to understand the molecular basis of TNFα-mediated induction of adhesion molecules in endothelial cells and provide insights to prevent and treat cardiovascular diseases caused by endothelial cell dysfunction.

## Data Availability

The raw data supporting the conclusion of this article will be made available by the authors, without undue reservation.
