# Supplementary material for: The crosstalk between neuropilin-1 and tumor necrosis factor-α in endothelial cells
Source: Front Cell Dev Biol. 2024 Jun 27;12:1210944. doi: 10.3389/fcell.2024.1210944 (PMC11236538; doi:10.3389/fcell.2024.1210944)
Supplement: Supplementary file 1 [file Presentation1.pdf]

Supplemental Figure 1

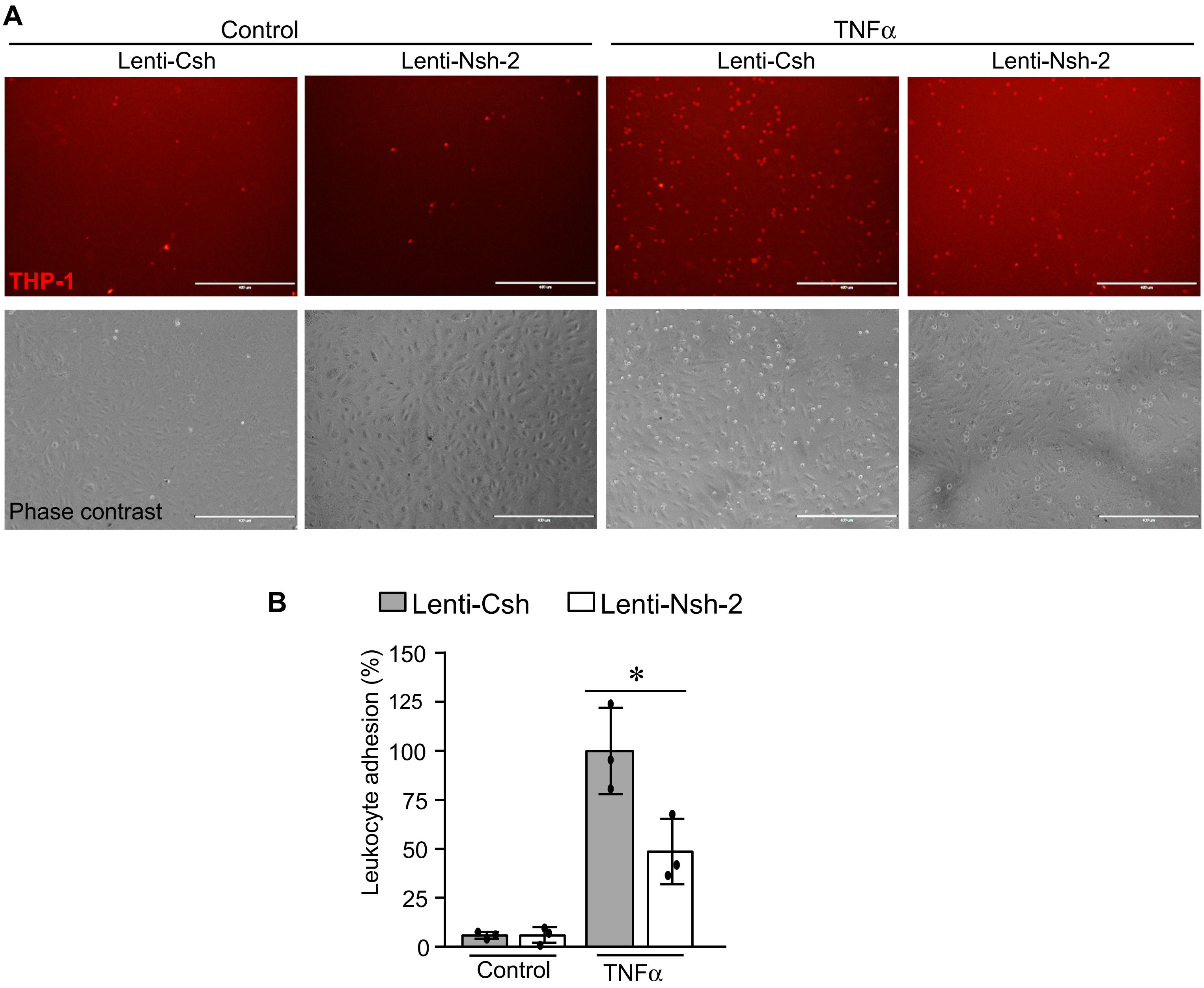

Supplemental Figure 1. Effect of Lenti-NRP1-shRNA#2 on leukocyte adhesion. HUVECs were infected with lentivirus expressing control shRNA (Lenti-Csh) and NRP1-shRNA-#2 (Lenti-Nsh-2), respectively, selected with puromycin for 48h, cultured to 80% confluence, and then stimulated with recombinant human TNFα (5 ng/mL) for 20 hours. CMTMR-labeled THP-1 cells were added to the cultured medium for 30 min and unbounded THP-1 cells were removed by gently rinsing with warm culture medium. Images were acquired using an EVOS cell imaging system. Representative images are from four to six images per group (A). The adhered THP-1 cells were manually counted and analyzed (B). *, $p<0.05$.

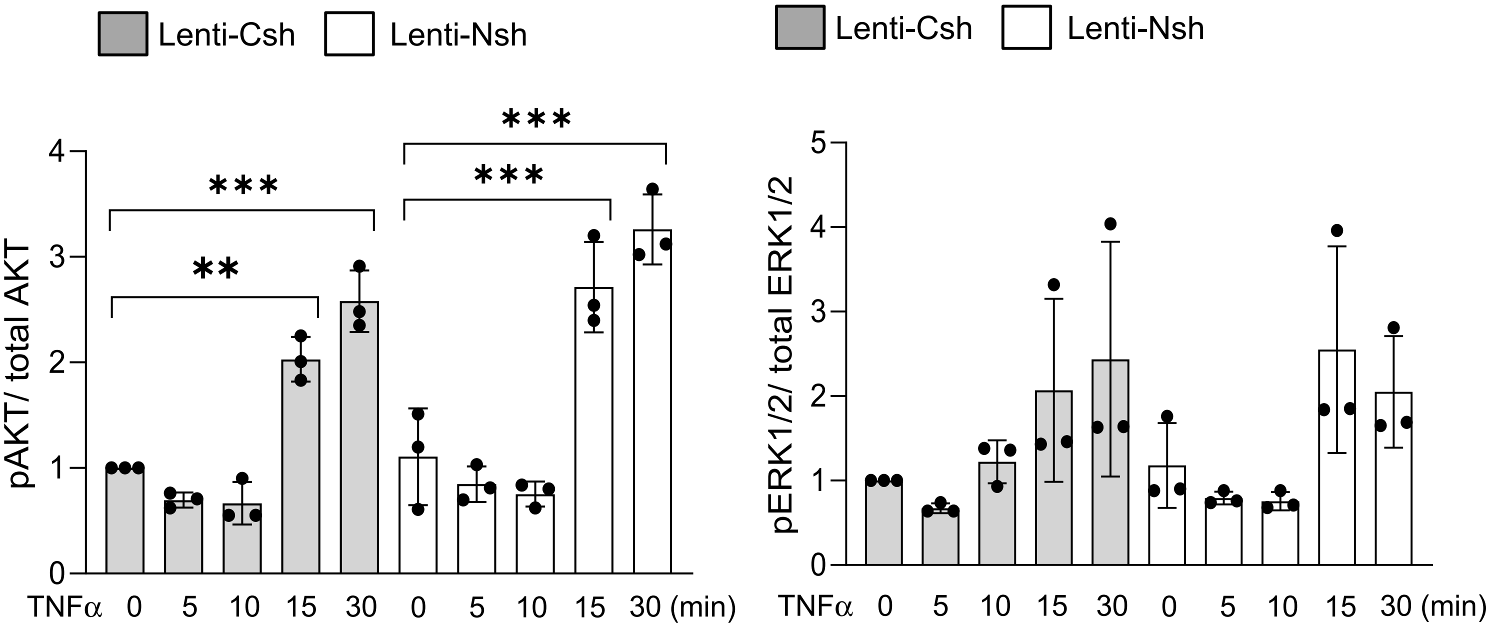

Supplemental Figure 2. Quantification of phosphorylated AKT and ERK1/2 in Fig.2A. Immunoblots shown in Fig.2A from independent experiments were analyzed with Image J and then compared. **, p<0.01, ***, p<0.001.

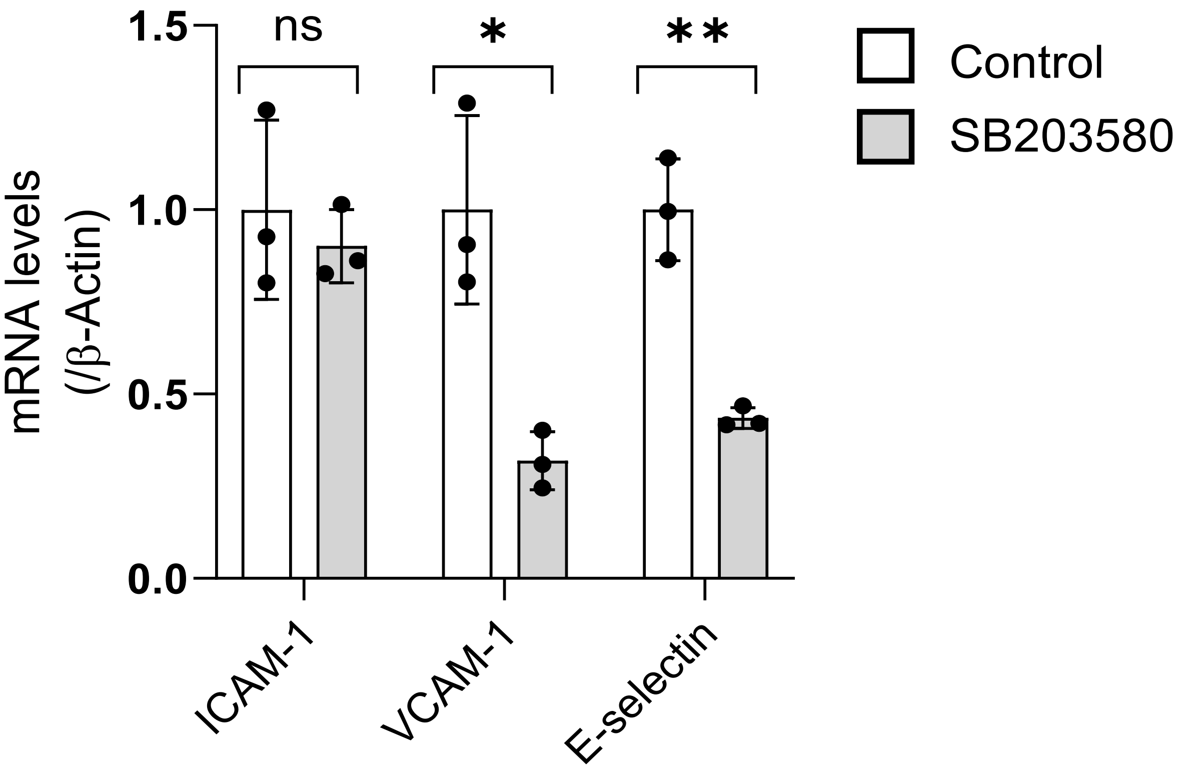

Supplemental Figure 3. Effect of p38 MAPK inhibitor SB203580 on adhesion molecules in NRP1 knockdown HUVECs. NRP1 knockdown human umbilical vein endothelial cells (HUVECs) were stimulated with recombinant human TNFα (5 ng/mL) for 20 hours and then subjected to qPCR. DMSO or SB203580 (10 μM) was added 1 h prior to TNFα stimulation. Ns, no significance, *, $p<0.05$, **, $p<0.01$.

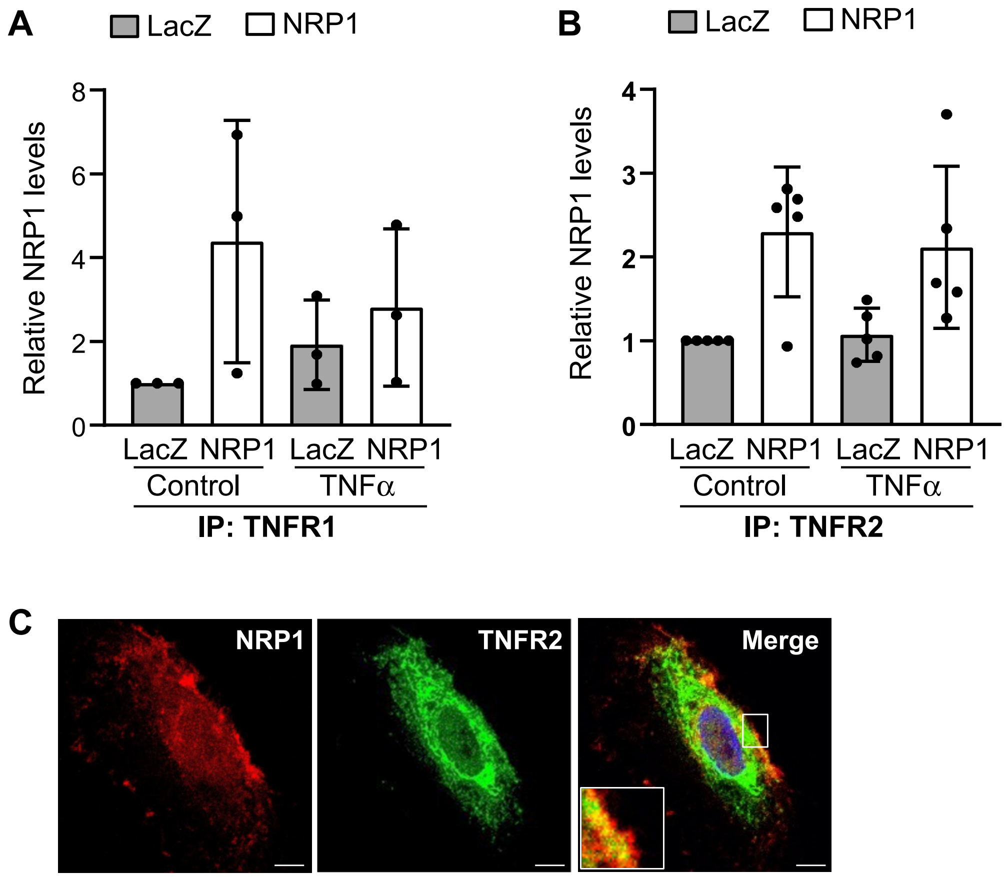

Supplemental Figure 4. TNFR-NRP1 interactions. A-B. Quantification of NRP1 levels in the immunoprecipitates in Fig.3B. Immunoblots shown in Fig.3B from independent experiments were analyzed with Image J and then compared. **C**. HUVECs were subjected to immunofluorescent staining with the indicated antibodies. Nuclei were counterstained with DAPI. Scale bar, 10 μm.

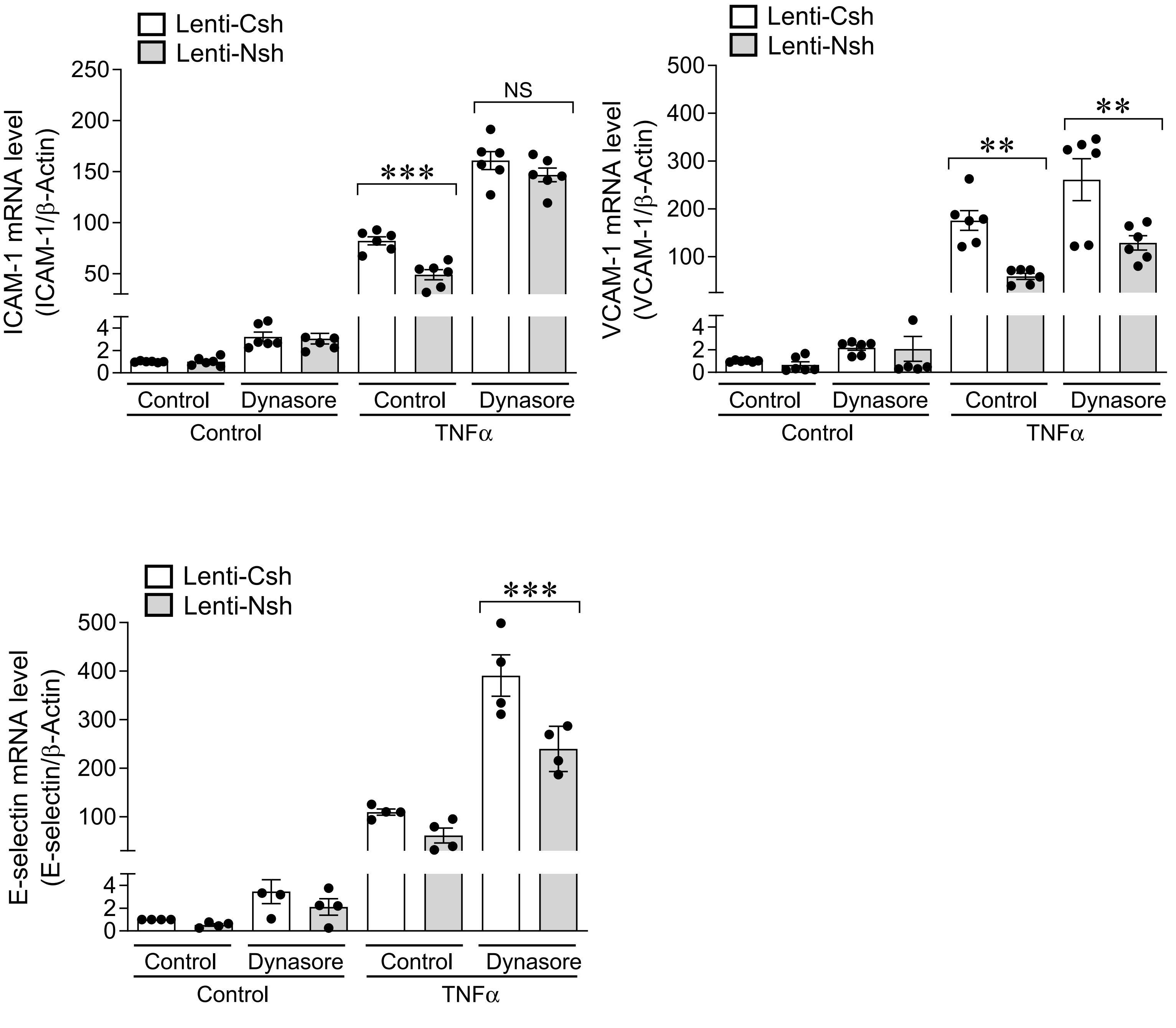

Supplemental Figure 5. Effect of endocytosis inhibitor Dynasore on expression of adhesion molecules in control and NRP1 knockdown ECs. Control and NRP1 knockdown HUVECs were pre-treated with Dynasore (40 μM) for 1h, stimulated with TNFα (5 ng/mL) for 20 h and then subjected to qPCR. NS, not significant, **, $p<0.01$, ***, $p<0.001$.

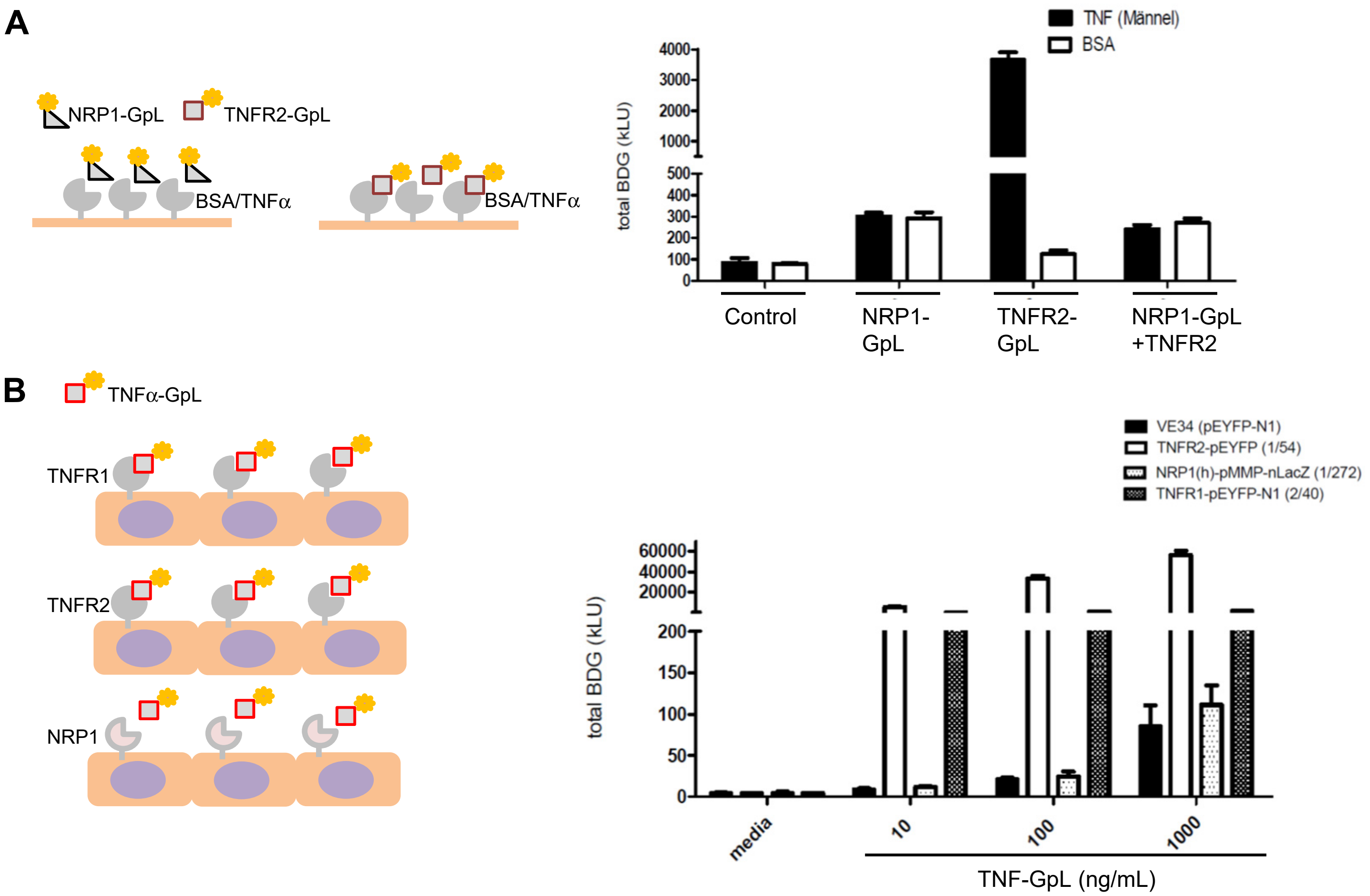

Supplemental Figure 6. No binding between NRP1-GpL and TNFα in microplate-based binding assay or cultured HEK293 cells. A. Microplates were coated with BSA and TNFα, respectively, and then incubated with NRP1-GpL and TNFR2-GpL, respectively. Luciferase activity was measured to quantified the retained NRP1-GpL and TNFR2-GpL, respectively. **B**. HEK293 cells were transfected with control plasmid and plasmids expressing TNFR1, TNFR2 and NRP1, respectively, and then incubated with TNFα-GpL at different doses. Luciferase activity was measured to quantify the binding of TNFα-GpL on cells.

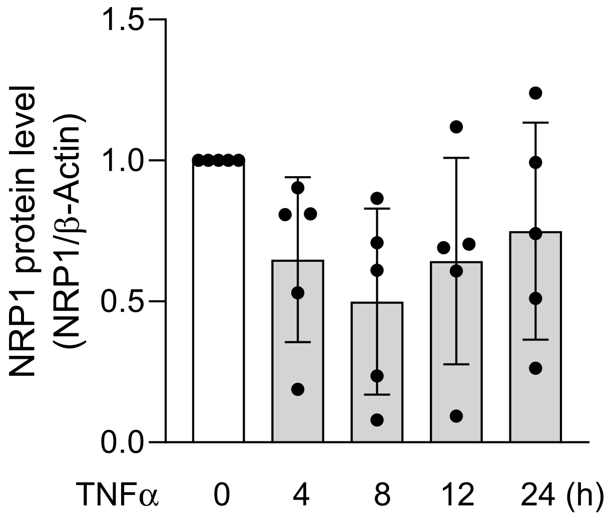

Supplemental Figure 7. Quantification of NRP1 levels in the Western blots in Fig.5A. Immunoblots shown in Fig.5A from independent experiments were analyzed with Image J and then compared.

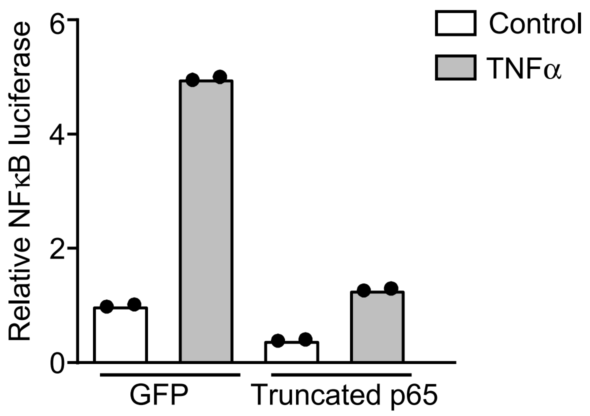

Supplemental Figure 8. Effect of mutant NFκB p65 overexpression on NFκB activation. HUVECs were first infected with adenovirus NFκB-reporter luciferase and then infected with adenovirus expressing mutant NFκB p65 or GFP as control. Luciferase activity was analyzed 24 h after TNFα stimulation.

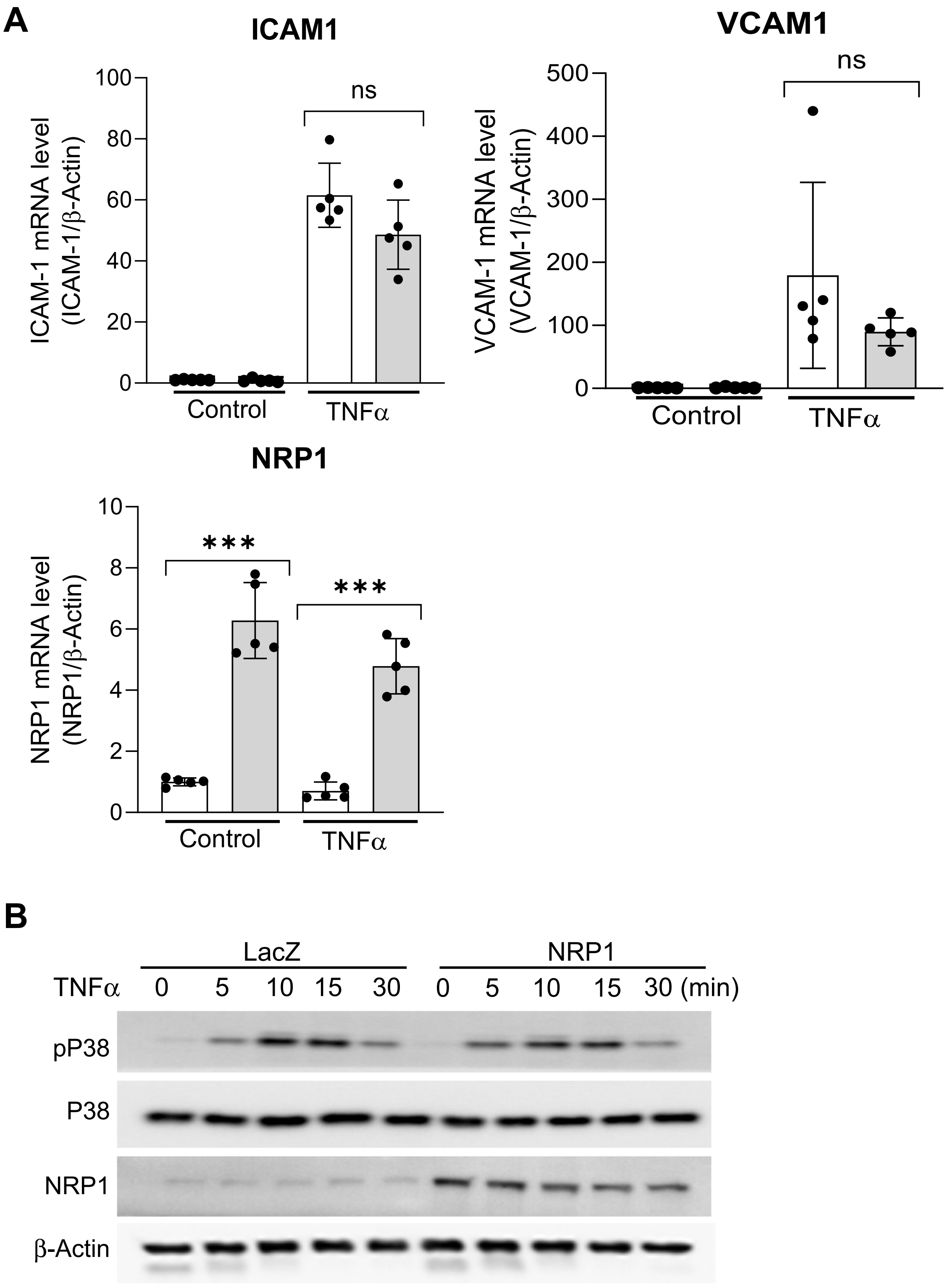

Supplemental Figure 9. Overexpression of NRP1 does not affect expression of adhesion molecules or phosphorylation of MAPK p38. HUVECs were infected with retrovirus expressing NRP1 or LacZ as a control, and then stimulated with TNFα (5 ng/mL) for 20 h (A) or the indicated times (B). qPCR and western blotting were performed, respectively. The blots are representative of 3 independent experiments. ***, $p<0.001$. ns, not significant.
